# Cancer Cell Mechanics: Adhesion G Protein-coupled Receptors in Action?

**DOI:** 10.3389/fonc.2018.00059

**Published:** 2018-03-13

**Authors:** Nicole Scholz

**Affiliations:** ^1^Rudolf Schönheimer Institute of Biochemistry, Division of General Biochemistry, Faculty of Medicine, University Leipzig, Leipzig, Germany

**Keywords:** adhesion G protein-coupled receptors, mechanobiology, cancer, cytoskeleton, extracellular matrix

## Abstract

In mammals, numerous organ systems are equipped with adhesion G protein-coupled receptors (aGPCRs) to shape cellular processes including migration, adhesion, polarity and guidance. All of these cell biological aspects are closely associated with tumor cell biology. Consistently, aberrant expression or malfunction of aGPCRs has been associated with dysplasia and tumorigenesis. Mounting evidence indicates that cancer cells comprise viscoelastic properties that are different from that of their non-tumorigenic counterparts, a feature that is believed to contribute to the increased motility and invasiveness of metastatic cancer cells. This is particularly interesting in light of the recent identification of the mechanosensitive facility of aGPCRs. aGPCRs are signified by large extracellular domains (ECDs) with adhesive properties, which promote the engagement with insoluble ligands. This configuration may enable reliable force transmission to the ECDs and may constitute a molecular switch, vital for mechano-dependent aGPCR signaling. The investigation of aGPCR function in mechanosensation is still in its infancy and has been largely restricted to physiological contexts. It remains to be elucidated if and how aGPCR function affects the mechanoregulation of tumor cells, how this may shape the mechanical signature and ultimately determines the pathological features of a cancer cell. This article aims to view known aGPCR functions from a biomechanical perspective and to delineate how this might impinge on the mechanobiology of cancer cells.

## Introduction

Cancer is an ongoing threat to human health. In 2012, worldwide 14 Mio people were diagnosed with a form of malignant cancer (1) and the International Agency for Research on Cancer predicts an increase in cancer cases up to terrifying 20 Mio by 2030 (2).

Each cell in our body has the capacity to morph into a tumor cell if its genome accumulates a critical load of lesions to disturb the delicate balance between proliferation and apoptosis. Cancer scientists have long been aware of genetic and epigenetic changes as well as alterations in the perception and integration of biochemical signals as the source of tumorigenesis and metastasis. However, more recently it has been recognized that cell growth, invasion and metastasis are intricately linked to the constituent cells’ facility to detect, integrate and respond to intrinsic and extrinsic mechanical cues (3). Since 2009 the National Cancer Institute has been supporting 12 leading institutions in the US to establish Physical Science—Oncology Centers and to build a collaborative network to interrogate how the micromechanical interior and exterior of a tumor cell shapes its biochemical properties and *vice versa*. Thus, the realization that every cancer cell is subject to similar physical constraints may bring forth an exciting era of cancer research and has the potential to revolutionize the development of therapeutics.

Adhesion G protein-coupled receptors (aGPCR) comprise the second largest group within the superfamily of G protein-coupled receptors (GPCRs) and are expressed in most organ systems. aGPCRs are known to modulate pivotal cellular functions including migration, adhesion, polarity and guidance (4). Consistently, members from all aGPCR subfamilies have been reported in the context of dysplasia and tumorigenesis in one way or another (5–8). While for some receptors it is mere evidence of faulty expression or changes in their activity (e.g., VLGR1/ADGRV, LPHN/ADGRL), for others more detailed knowledge about the molecular mechanisms that make cells or tissues go awry have emerged [e.g., CD97/ADGRE5 (9–11), GPR116/ADGRF5 (12, 13), GPR133/ADGRD1 (14, 15), GPR56/ADGRG1 (16–19), and BAI1/ADRGB1 (20–22)].

**Figure 1:**
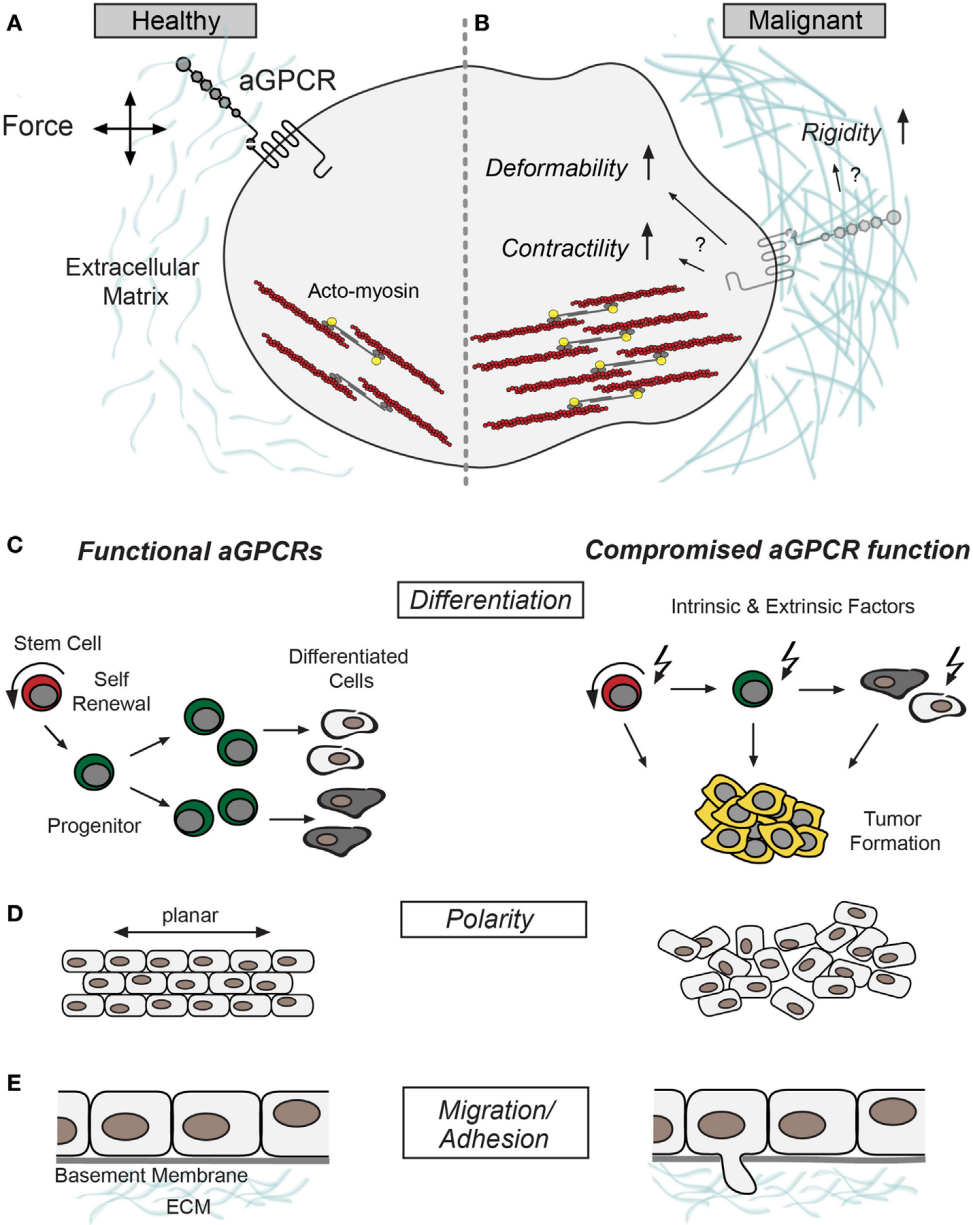
Putative roles of adhesion G protein-coupled receptors (aGPCRs) in cancer. **(A)** Schematic illustration of a healthy cell without structural abnormalities and proper aGPCR function aligned with **(B)** a malignant cell signified by increased contractility, compliance, and extracellular matrix rigidity (28). These changes could intersect with aGPCR dysfunction and/or altered expression. Several aGPCRs have been shown to signal through RhoA (18, 29), which indirectly stimulates myosin-light chain phosphorylation (yellow) to promote acto-myosin contractility (F-actin and myosin in red and dark gray, respectively) (30). **(C–E)** Show different cellular properties previously associated with aGPCR function and malignancies; left panel: intact aGPCR function. Right panel: cellular deficits related to compromised aGPCR function/expression. **(C)** Schemes of differentiation hierarchy. Adult stem cells (indicated in red) renew themselves and produce progenitor cells (indicated in green), which go through several rounds of division before they differentiate into specialized cells (31). Compromised aGPCRs may promote a shift from differentiation to proliferation, which may cause progenitor cell expansion and benign or malignant tumorigenesis (right). **(D)** Cell and tissue polarity is fundamental for oriented cell division and tissue formation, processes that have been associated with aGPCR function (32–34). **(E)** aGPCR malfunction often results in phenotypes linked to defective cell migration (35–38). Defective cell-cell and cell-matrix adhesion of tumor cells may be causally associated with aGPCR dysfunction and catalyzes tumor cell migration and invasion.

A more recent development in aGPCR biology is the finding that these receptors possess mechanoceptive features (23–27), which contrasts the common view of GPCRs as chemosensors. Although most of the data that underpin this finding have been collected in physiological settings, it is conceivable that many of the seemingly unrelated pathophysiological phenotypes caused by aGPCR malfunction may be the result of defective mechanosensation. Thus, aGPCRs may constitute a connecting element between neoplastic, malignant, or metastatic properties and the mechanical phenotype of a given cell (Figure 1). This article aims to connect the current knowledge of aGPCR biology and cancer physics and will discuss potential intersections.

## Mechanical Signature and Malignancy

En route from healthy over malignant to a metastatic state a cell undergoes an arduous ‘force journey’ signified by a mechanical reciprocity between cell contractility and extracellular matrix (ECM) rigidity. In other words, the biophysical signature of a cell and its ECM generates a lively biomechanical ‘conversation’ in which cellular contractile forces that impinge on the ECM counterbalance the elastic resistance of the ECM toward this mechanical stimulus (39).

To detect, integrate and react to mechanical inputs nature has invented an interconnected hierarchy of mechanical systems build from force sensors, adhesive molecules, focal adhesions as well as cytoskeletal and filamentous elements. Together these components comprise the molecular backbone of this delicate force balance between cells and their ECM, crucial for numerous cellular properties as well as the development and maintenance of tissues.

Dysplasia often leads to the generation of aberrant force values, which throw the system off balance leading to changes in the cytoskeletal and filamentous cellular architecture (40). An intriguing example of this phenomenon is the exchange from keratin-based to vimentin-based cytoskeleton in mammary tissue, which constitutes an indicator for epithelial-to-mesenchymal transition (EMT) (41–43). As malignancies progress cell and ECM feature specific changes common in many cancer types (Figures 1A,B): (i) The ECM exhibits increased stiffness (28). In breast malignancies, for example, this seems to be due to an integrin-dependent increase in collagen cross-linking and tissue fibrosis (44, 45). (ii) The ECM is remodeled due to the release of proteolytic enzymes (e.g. matrix metalloproteases) from protrusive cell processes (46, 47). (iii) Tumor cells show reduced adhesiveness to adjacent tumor and non-tumor cells, caused by loss in cell–cell contact integrity (48–51). (iv) Tumor cells often display increased compliance/deformability and contractility (52–55). This way metastatic cells are structurally fine-tuned to break through basal laminae, squeeze through tissues, and form secondary tumors at distant sites. Finally, elevation in ECM rigidity and cell contractility is attended by an increase in malignancy and metastatic potential (28, 56). Importantly, these cellular and extracellular anomalies manifest in many cancer types and appear as a prerequisite for cells to succumb to mechanical insults associated with dysplasia and the metastatic cascade.

## Molecular Components and Mechanical Phenotype

According to the National Cancer Institute more than 30 tumor markers are currently used to diagnose and help manage different cancer types (https://www.cancer.gov/about-cancer/diagnosis-staging/diagnosis/tumor-markers-fact-sheet, accessed December 10, 2017). In light of the progress in genomic profiling technologies and tailor-made molecular targeting tumor markers will take an increasingly important role. However, to date we understand surprisingly little about the biological rationale behind their misexpression let alone the cellular signaling routes they employ and interactions they engage in to control tumor growth, invasion and metastasis. To this end it will be particularly interesting to decipher molecular players responsible for the mechanical metamorphosis of tumors. Several molecules have been documented in this context including constituents of focal adhesion complexes (FA), cytoskeletal and filamentous components as well as cell adhesion molecules (CAMs). Here, I will focus on FA protein complexes as mechanosensing entities and particularly integrin receptors, as they constitute *bona fide* mechanotransducers with intricate ties to the evolution of the malignant phenotype.

FAs comprise integrin-based macromolecular assemblies that physically link the cell’s exterior substrate and interior cytoskeleton. Integrins nucleate a plethora of molecules at the intracellular interface to build highly dynamic biochemical signaling hubs key to several cellular aspects including cell shape, proliferation, survival, differentiation, and motility (57–59). The composition and maintenance of FAs relies on the local force profile generated by myosin-driven contraction of the cytoskeleton (‘inside-out’) or by perturbations of the ECM (‘outside-in’) (60–62). The core of cellular FA mechanotransduction are integrins (63, 64). They act as direct and indirect mechanotransducers by reacting to mechanical force either with conformational changes or with aggregation to build a platform for force-transfer to the cytoskeleton, respectively. Application of external forces or changes in ECM stiffness stimulate integrins to activate RhoGTPase and Rho-associated kinase, which modulates myosin-light-chain phosphorylation (30). This way cell contractility increases further escalating ECM stiffness through aberrant integrin-signaling, collagen deposition and cross-linking (45). This deadly, self-sustaining positive feedback loop is intricately intertwined with Erk-dependent mitogenic signaling, which controls cell proliferation and the development of mammary cancer (44, 45). Moreover, due to their ability to regulate expression and activity of metalloproteases integrins play their part in invasion and metastasis (65, 66).

Among the canonical signaling routes triggered by integrins, the activation of the focal adhesion kinase (FAK) was shown to play a major role in several cancer types. FAK shapes the structure and function of FAs, e.g., through α-actinin-dependent changes in F-actin crosslinking and/or RhoGTPase-driven modulation of actomyosin contractility (67–69). Interestingly, a recent computational study advocates a novel mechanoenzymatic mode in which FAK localizes to PIP_2_ enriched FA-membranes to directly sense membrane perturbations that in turn induce specific conformational changes nourishing Ras-mediated delivery to the nucleus (70, 71).

Integrins have been in the limelight of mechanotransduction in cancer. However, nature has equipped cells with different membrane receptors to comply with cell-specific demands and to ensure reliable mechanotransduction. Hence, it is more likely than not that cancer cells utilize additional sophisticated *bona fide* force sensors.

## Adhesion-GPCRs: Mechanotransduction and Malignancy

The aGPCR family counts 33 mammalian homologs serving functions in a multitude of developmental and physiological phenomena (4, 72). aGPCRs possess a modular architecture composed of an extracellular domain (ECD), a seven-transmembrane-spanning (7TM) domain and a intracellular domain (72). Their ECDs are exceptional in size and complexity generally imparted by the presence of various adhesive structural folds, which promote interactions with ECM constituents and receptors of adjacent cells (72). The ECDs of most aGPCRs contain a GPCR autoproteolysis-inducing (GAIN) domain (73), which usually includes a canonical GPCR proteolysis site (GPS; H/R_−2_–L/M/I_−1_ ↓ T/S/C_+1_) that catalyzes self-cleavage of the receptor (74–77). Once the resulting N- and C-terminal fragments (NTF and CTF) are delivered to the cell surface they reunite through a non-covalent bond to build heterodimeric receptor molecules (73). A seminal discovery with respect to aGPCR signaling was the identification of a short amino acid sequence C-terminal of the GPS motif as a cryptic tethered ligand (*Stachel* sequence) with agonistic capacity (78, 79). However, how the *Stachel* is freed from the envelop of the GAIN domain pocket it resides in, and whether receptor cleavage is involved is matter of ongoing debate (79, 80). Thus, while the functional relevance of GAIN/GPS-mediated cleavage remains in large part controversial, the GAIN domain in juxtaposition to the 7TM has become the defining aGPCR signature (72).

Another unifying property of aGPCRs may be their mechanosensitivity (23–27, 81). For example, GPR56/ADGRG1 was suggested to control muscle fiber size through its stimulation in response to mechanical preload (23). In *Drosophila*, LPHN/CIRL/ADGRL tunes the mechanosensitive profile of sensory neurons through cAMP-dependent modulation of ionotropic receptors (25, 80). EMR2/ADGRE2 was the first aGPCR, for which a mechano-dependent malfunction was directly linked to disease. Vibratory uticaria causes hives and systemic manifestations in patients when challenged with cutaneous vibration. The underlying cause is a single point mutation in the GAIN domain of EMR2, which might destabilize the NTF-CTF heterodimer to enhance receptor activity and induce histamine release from mast cells (27).

Physical anchorage constitutes a logical prerequisite for mechanosensing as it enables measurement of movements relative to the sensor-bearing structure to convey force information. Thus, attachment and size-dependent tensility of NTFs may be critical for mechanosensing through aGPCRs. NTF-size and domain layout vary across the receptor family. Moreover, untypical for canonical GPCRs, aGPCRs are vividly spliced producing a palette of differentially sized NTFs (74, 82, 83), which adds an additional level of structural flexibility and functional complexity. While the underlying reasons for this variability are unclear it is tempting to speculate that aGPCR-NTFs are built to sense chemical or mechanical signals from the ECM, bridge intercellular gaps or even read out the dimension of these gaps. The NTF size of each receptor may thus be tuned to meet the specific geometric demands of its expressing tissue enabling reliable and precise distance sensing and mechanotransduction. This notion is in line with the set-point of neuronal mechanosensitivity conferred by *Drosophila* CIRL, which inversely scales with the length of its NTF as shown by artificial elongation experiments (80). aGPCR function in diverse biological processes such as the establishment of cell and tissue polarity (32–34), synaptogenesis (84–86), and myelination of peripheral nerves (24) favors a palette of alternative aGPCR variants generated through transcriptional modifications.

The newfound mechanoceptive facility of aGPCRs in combination with cellular functions and implications in cancer is particularly daunting in light of the perilous force journey cells go on as they transform, turn malignant and potentially become metastatic (Figures 1C–E).

GPR56 presents one of the most well-studied aGPCRs with respect to cancer progression. In melanoma cells, GPR56 curtails cell growth and metastasis indicative of its suppressive function in tumorigenesis (16). While this phenotype was associated with PKCα/VEGF signaling-based defects in angiogenesis (17), other cellular routes, e.g., Gα_12/13_/RhoA seem to supplement the GPR56-dependent signaling profile in melanoma cells (18). Rho-dependent signaling plays roles in several steps of cancer progression (30), most of which can be traced back to its ability to control contractility, stress fiber formation and FA reinforcement (67); properties that confer cell migration through a heterogeneous stromal environment. This is in agreement with GPR56’s location at the leading edge of membrane filopodia and its colocalization with α-actinin in human glioma cells (87).

Recent work revealed that mesenchymal differentiation and radioresistance, defining features of glioblastoma, are repressed by GPR56 (19). The authors propose a model in which GPR56 obstructs nuclear factor kappa alpha (NF-κα) to inhibit mesenchymal differentiation. During differentiation, however, NF-κα levels are potentiated due to tumor (TNFα)-mediated activation and relief of GPR56 suppression. Atomic force microscopy of human colon cancer cells (HCT116) uncovered that TNFα-induced EMT is indeed associated with cytoskeletal rearrangements and changes in cell elasticity (88). Contrasting the situation in melanoma and glioblastoma, a recent *in vivo* study uncovered GPR56 as a novel marker for leukemic subpopulations signified by high repopulation capabilities typical for acute myloid leukemia (89). Interaction of leukemic stem cells and bone marrow niches is the leading cause of repopulation capacity and acute myloid leukemia-relapse. One hypothesis suggests that adhesive molecules [e.g., integrins (90), aGPCR (89)] retain leukemic stem cells in the niche, sheltering them from chemotherapy.

Brain angiogenesis inhibitor-1 (BAI1/ADGRB1), another cancer-associated aGPCR, is enriched in the brain and has been associated with tumor angiogenesis (8). In this process tumors induce vessel formation essential for oxygen and nutrient supply (91). Separation of BAI1’s NTF at the GPS results in the release of a fragment, vasculostatin-120, which interferes with angiogenesis through the inhibition of endothelial cell proliferation (92) and microvascular endothelial cell migration (21). Soluble BAI1 was also shown to ablate angiogenesis and glioma growth *in vivo* (20, 93–95). The BAI1-NTF is composed of one Arg-Gly-Asp integrin-binding motif (RGD) and five thrombospondin type 1 repeats. Interestingly, another functionally active fragment, vasculostatin-40, breaks away from the BAI1-NTF to mediate antiangiogenic effects *in vitro* and *in vivo* (94, 96). Thus, BAI1 N-termini negatively regulate vessel formation through a cell non-autonomous signaling mode. In contrast, ELTD1/ADGRL4 possesses proangiogenic capacities and is believed to modulate vascular sprouting (97). In glioblastoma, for example, ELTD1 levels are elevated and have been shown to modulate glioma growth (97–99). Interestingly, loss of *Eltd1* in mice augments cardiac hypertrophy in response to pressure preload (100), which might indicate mechano-dependence of ELTD1 function in angiogenesis.

The Ingber lab established an intriguing model describing the mechanical control of angiogenesis (101). This model is based on the notion that local thinning of the ECM increases its compliance and causes local cell distortion generated by tractional forces of surrounding cells. Subsequently, integrin-dependent force transfer across the cell membrane alters the biochemical machinery to drive coordinated cell growth, motility and ultimately capillary patterning (102–106). This mechanical force concept applies to tumor angiogenesis as well, only here the ECM stiffens to shift the force balance. Strikingly, the anti-angiogenic potential of BAI1 seems to depend on the interaction of its soluble NTF with surface receptors (integrins and CD36) (92, 107). This begs the question whether BAI1 interacts with integrins to shape angiogenesis mechano-dependently.

BAI1 is downregulated in many forms of cancer (8), which may be due at least in part to gene silencing and somatic mutations (5, 95, 108). However, is it possible that cancer formation induces aberrant BAI1 expression after protein processing and trafficking? Could ECM stiffness break off the NTF on tumor cells to induce constitutive signaling and/or receptor internalization? BAI1 is known to direct cytoskeletal changes through direct and indirect signaling paths with PDZ-binding molecules as well as Rho and Rac molecules, respectively (109, 110). Hence, force-dependent misregulation of BAI1 could intersect with alterations in the cellular mechanical signature.

Here GPR56, BAI1, and ELTD1 serve to illustrate the putative role of aGPCRs in cancer cell mechanics. However, various aGPCRs have been associated with cancer development and progression including GPR133/ADGRD1, which is required for glioblastoma growth (14); GPR116/ADGRF5, which furthers breast cancer metastasis (12); and CD97/ADGRE5 seems to enhance tumor cell invasion in several human malignancies (111–114).

## Integrins and aGPCRs

The force-sensing capabilities of tumor cells can be in large part traced back to integrins. Interestingly, several aGPCRs have been shown to interact with integrins; often by NTF shedding and cell non-autonomous signaling at ectopic sites. For example, soluble CD97/ADGRE5 was shown to interact with endothelial α5β1 and αvβ3 integrins to promote angiogenesis *in vivo* (9). The NTF of GPR124/ADGRA2 is released from cultured endothelial cells during angiogenesis and engages with ECM glycosaminoglycans. Subsequently, the NTF is truncated in a second cleavage event exposing a RGD motif to promote endothelial cell survival by interconnecting integrin αvβ3 and glycosaminoglycans (115). However, GPR124 was shown to modulate Wnt signaling to shape developmental CNS angiogenesis *in vivo* RGD motif-independently (35, 116, 117). In contrast, neuronal GPR56 and integrin α3β1 interact to regulate cell migration and cortical development in a cell autonomous manner (118). Collectively, the available data suggests a model in which aGPCRs engage in a *cis* or *transcellular* conversation with integrins. Moreover, aGPCRs may use RGD motifs as an anchor to target soluble aGPCR-NTFs to distant sites (9, 115, 119).

As aGPCRs function as sensors and probably transducers of mechanical information it seems plausible that they perform next to or in conjunction with integrins to shape the biophysical signature of tumor cells. Several properties of aGPCR are in line with this notion: (1) aGPCR interact with ECM molecules and are involved in the coordination of cytoskeletal architecture indirectly through G-protein, Rho and Rac dependent signaling cascades and directly, e.g., *via* PDZ proteins (120). Thus, similar to integrins aGPCRs may connect extracellular ECM and intracellular cytoskeleton. (2) aGPCR promote bidirectional signaling (119). (3) aGPCRs have the capacity to dimerize or oligomerize (121, 122). Thus, it is enticing to speculate that aGPCRs constitute novel mechanotransducers that shape the biomechanical profile of cells in health and disease.

## Conclusion

Adhesion G protein-coupled receptors shape a number of cellular aspects intricately tied to tumorigenesis (Figures 1C–E), which is why they might hold tremendous potential as molecular targets for the development of novel therapeutic strategies to control cancer cell plasticity and malignancy. However, aGPCR research is still in its infancy and we are far from understanding their general roles in cancer and mechanobiology. Thus, it will be interesting to monitor putative aGPCR-dependent changes in the cytoskeleton during transformation and beyond using super-resolution and live imaging methodologies. Moreover, a complementary approach employing atomic force microscopy and subcellular laser ablation will help to analyze viscoelastic changes of malignant and metastatic cells with respect to loss or dysfunction of aGPCRs. Finally, this knowledge might contribute to a more comprehensive understanding of aGPCRs’ roles as mechanical force sensors in physiological and pathophysiological settings.

## Author Contributions

NS wrote and revised/edited the manuscript; prepared the figure and approved the article for publication.

## Conflict of Interest Statement

The author declares that the research was conducted in the absence of any commercial or financial relationships that could be construed as a potential conflict of interest.
